# PSYCHOLOGICAL PREDICTORS OF FALLS EFFICACY AMONG FILIPINO OLDER ADULTS IN AN ELDERLY DEVELOPMENT PROGRAM

**DOI:** 10.1093/geroni/igz038.232

**Published:** 2019-11-08

**Authors:** Godfrey Josef R Torres, Katrina Marie Jolejole, Nephtaly Joel Botor, Teri-Marie Laude

**Affiliations:** University of the Philippines - Los Banos, Los Banos, Laguna, Philippines

## Abstract

Falls, common among aging persons, typically lead to catastrophic health consequences. Studies show several factors influencing an older person’s risk to falling. Depression, a psychological condition, was identified as one of these factors. With the goal of determining potential psychosocial interventions for older persons, the present study explores what other psychological variables may explain falls efficacy, i.e., perceived concern about falling. 81 older adults who were participants in an elderly development program answered a socio-demographic survey and several scales (i.e., Satisfaction with Life Scale, Flourishing Scale, Geriatric Depression Scale – Short Form, Falls Efficacy Scale – International) to measure falls efficacy and other psychological variables. Bivariate correlation revealed that falls efficacy significantly increases as family problems (r=.228, p=.045), health concerns (r=.231, p=0.040), financial difficulties (r=.345, p=.002), and depression (r=.403, p<.001) increase. Conversely, it significantly decreases as psychological well-being (r=-.255, p=.022) and perceived resilience (r=-.459, p<.001) decrease. Multiple regression analysis confirmed that while depression is a significant positive predictor, F (1,79)=15.31, p<.001, R=.403, explaining 16.2% of falls efficacy variance, anxiety-provoking situations (i.e., family problems, health worries, financial worries) also explain additional falls efficacy (6.6% variance), F (4, 71)= 5.234, p<.001, R=.477, wherein financial worry is a significant positive predictor. Furthermore, entering psychological well-being and resilience in the model adds an additional variance of 6.6%, F (6, 69)=4.533, p<.001, R=.532, but only resilience is a significant negative predictor. This paper culminates with recommendations on potential research on the psychosocial dimension of falls and possible interventions to mitigate falls among older persons.

